# Research on Obesity: A Quarter-Century Experience from the Tehran Lipid and Glucose Study

**DOI:** 10.5812/ijem-168110

**Published:** 2026-01-31

**Authors:** Sara Sadeghi, Behnaz Abiri, Farhad Hosseinpanah, Majid Valizadeh, Fereidoun Azizi, Maryam Mahdavi, Maryam Barzin

**Affiliations:** 1 Obesity Research Center, Research Institute for Metabolic and Obesity Disorders, Research Institute for Endocrine Sciences, Shahid Beheshti University of Medical Sciences, Tehran, Iran; 2 Obesity Research Center, Research Institute for Endocrine Sciences, Shahid Beheshti University of Medical Sciences, Tehran, Iran; 3 Endocrine Research Center, Research Institute for Endocrine Disorders, Research Institute for Endocrine Sciences, Shahid Beheshti University of Medical Sciences, Tehran, Iran

**Keywords:** Tehran Lipid and Glucose Study, TLGS, Obesity, Cohort Studies, Cardiometabolic Risk Factors, Iran

## Abstract

**Context:**

This paper aims to synthesize a quarter-century of research on obesity from the Tehran Lipid and Glucose Study (TLGS), a large prospective population-based cohort.

**Evidence Acquisition:**

A comprehensive literature search identified original studies from the TLGS investigating obesity indices, body composition, obesity phenotypes, and their associations with cardiometabolic outcomes.

**Results:**

Tehran Lipid and Glucose Study findings demonstrate alarming increases in the prevalence and incidence of general and abdominal obesity among Tehranian adults and children. The studies have established population-specific anthropometric cut-offs, revealed the complex associations of various obesity phenotypes with type 2 diabetes, cardiovascular disease, and chronic kidney disease, and highlighted the critical role of metabolic health over weight status alone.

**Conclusions:**

This substantial body of evidence provides crucial insights for crafting effective public health strategies and future research in transitioning populations.

## 1. Context

Obesity represents a critical global public health challenge, serving as a significant determinant in the development of non-communicable diseases (NCDs) such as cardiovascular disease and type 2 diabetes (T2DM) (1). The clinical definition of obesity is evolving beyond Body Mass Index (BMI). A recent global Commission on Clinical Obesity has proposed a major redefinition, emphasizing that the diagnosis should be based on the presence of clinical signs and symptoms of impaired organ function attributable to excess adiposity, rather than on BMI alone. This new framework introduces two diagnostic categories: "Clinical Obesity," characterized by objective adiposity-related health impairments (e.g., breathlessness, joint pain, metabolic dysfunction), and "Pre-clinical Obesity," defined by the presence of excess adiposity without current organ dysfunction but with increased future disease risk. This paradigm shift underscores the critical need to use adjunct measures such as waist circumference (WC), waist-to-hip ratio (WHR), or direct body composition assessment for a more accurate diagnosis and risk stratification (2). The escalating prevalence of obesity, driven by obesogenic environments, is particularly alarming in the Middle East and North Africa region, which has experienced rapid socioeconomic and nutritional transitions. As a key nation in this region, Iran has witnessed a dramatic epidemiological shift, with NCDs now constituting the primary cause of mortality and morbidity (1, 3, 4). Therefore, understanding the dynamics of the obesity epidemic within this specific context, through the lens of both traditional and contemporary diagnostic criteria, is of paramount importance for developing targeted interventions. Developing comprehensive strategies to combat obesity requires robust, population-based longitudinal data to elucidate its determinants and long-term consequences. To address this fundamental need in Iran, the Tehran Lipid and Glucose Study (TLGS) was established in 1999. This prospective cohort study was designed to investigate the prevalence, incidence, and risk factors of NCDs, with the ultimate goal of developing cost-effective prevention and control strategies. With over two decades of follow-up, the TLGS provides an invaluable repository for tracing the evolution of the obesity epidemic (3, 4). The TLGS was launched as the first large-scale, population-based prospective cohort in Iran, initially enrolling a representative sample of 15,005 residents from Tehran. Its methodology includes periodic examinations every three years, collecting comprehensive data on demographics, lifestyle, anthropometrics, and biochemical parameters. A distinctive feature is its integration of a community-oriented lifestyle intervention program. Over more than two decades, the TLGS has established itself as an indispensable national resource for researching NCDs and their risk factors (3, 4). The study's longitudinal data have been collected in successive phases at approximately 3-year intervals: Phase I (1999 - 2001, baseline), phase II (2002 - 2005), phase III (2006 - 2008), phase IV (2009 - 2011), phase V (2012 - 2015), phase VI (2016 - 2019), and phase VII (2020 - 2022). Each phase involves a comprehensive re-examination of the cohort, allowing for the assessment of trends, incidence, and the long-term natural history of conditions such as obesity and its cardiometabolic consequences. Over the past quarter-century, the TLGS has been a pivotal platform for extensive research into obesity. This article synthesizes this vast experience by reviewing key findings on the rising trends of general and abdominal obesity, establishing population-specific body composition thresholds, and elucidating the complex associations between diverse obesity phenotypes — such as metabolically healthy obesity — and the incidence of cardiometabolic disorders, including cardiovascular disease (CVD), T2DM, and chronic kidney disease. The review aims to translate these findings into evidence-based lessons for public health practice and future research.

## 2. Evidence Acquisition

To compile the relevant literature, a systematic search of major electronic databases was undertaken for this review, including PubMed/Medline, Scopus, and Web of Science, from inception. The search strategy incorporated keywords and MeSH terms for concepts including "obesity," "overweight," "body composition," "cardiometabolic risk factors," "cohort studies," and "Tehran Lipid and Glucose Study" OR "TLGS." No restrictions were placed on publication language. After removing duplicates, the titles and abstracts were assessed for relevance through screening. Subsequently, a full-text assessment of the potentially eligible articles was performed using predefined inclusion criteria, focusing specifically on original research articles from the TLGS that investigated obesity indices, body composition, obesity phenotypes, and their associations with cardiometabolic outcomes. This systematic approach ensured the identification and synthesis of all key evidence generated by the TLGS on obesity over the past quarter-century.

## 3. Results

### 3.1. Epidemiology of General and Abdominal Obesity

The following series of studies on the adult population within the TLGS cohort elaborate on the significant increases in general and abdominal obesity by investigating the dynamics across different age groups, incidence, and associated risk factors. Evidence from a decade-long prospective cohort study among Tehranian adults reveals alarming and sustained increases in the prevalence of both general and abdominal obesity. Findings from the TLGS demonstrated that the crude prevalence of obesity rose from 23.1% to 34.1%, while abdominal obesity saw a more dramatic surge from 47.9% to 71.1% over a 10-year follow-up period. Generalized estimating equation models confirmed these upward trends, indicating significant increases in the relative risk for both conditions in both genders, with a more pronounced rise observed in men and the youngest age group (20 - 39 years). Critically, these escalating trends were consistent across all sociodemographic subgroups, irrespective of marital status or educational level, underscoring a pervasive public health challenge that necessitates comprehensive, population-wide preventive strategies (5).

In another representative cohort of 10,045 Tehranian adults aged ≥ 19 years (4,480 men; mean age, 42.2 ± 15.1 years, and 5,565 women; mean age, 39.8 ± 13.8 years) followed from 1999 to 2017, the overall prevalence of severe obesity rose markedly from 4.6% (1.8% in men and 6.7% in women) in 1999 to 10.1% (4.7% in men and 14.3% in women) in 2017. The increasing trend persisted across both genders in the youngest age group, with the most pronounced rise observed among women aged 19 - 29 years, while the prevalence plateaued at older ages and remained stable among men aged over 50 years. After age- and gender-standardization using data from the Tehranian urban population, the prevalence of severe obesity was estimated at 1.9% in men and 5.7% in women in 1996, increasing to 4.5% and 10.9%, respectively, by 2016 (6).

Furthermore, based on a prospective cohort study conducted within the TLGS, the incidence and risk factors of obesity were investigated among 7,257 non-obese Tehranian adults (aged ≥ 20 years) over a median follow-up of 8 years. The cumulative incidence of obesity was 31.3% overall, with significantly higher rates in women (38.1%) than in men (23.4%). The highest incidence rates occurred in women aged 40 - 49 years (47.5 per 1000 person-years) and in men aged 20 - 29 years. Key predictors of obesity included higher baseline BMI (HR up to 83.9 in women), elevated waist circumference (HR = 5.4 in men, 2.4 in women), metabolic syndrome (HR = 2.4 in men, 1.7 in women), and lower educational level. Being married was also a significant risk factor, particularly for women and for men within the first 5.5 years of follow-up. No significant associations were found for physical activity or smoking (7).

In addition, in a 6-year cohort study within the TLGS, Barzin et al. (2018) (8) investigated the incidence and risk factors of abdominal obesity (AO), defined by sex-specific WC cut-offs, among 5,044 non-obese Iranian adults (mean age 37.7 years). The findings revealed an alarmingly high incidence of AO, with a cumulative incidence of 76.0% overall, which was significantly higher in men (83.6%) than in women (70.9%). The incidence rate was 138.7 per 1000 person-years in men, peaking in their 30s, compared to 77.1 in women, peaking in their 50s. Key risk factors for developing AO included having a dysmetabolic state (e.g., elevated blood pressure, glucose, or triglycerides) and a higher baseline BMI in both sexes. The analysis also identified distinct sex-specific predictors: Higher educational level and lower physical activity were risk factors in men, whereas married status was a significant risk factor in women (8).

**Figure 1.**
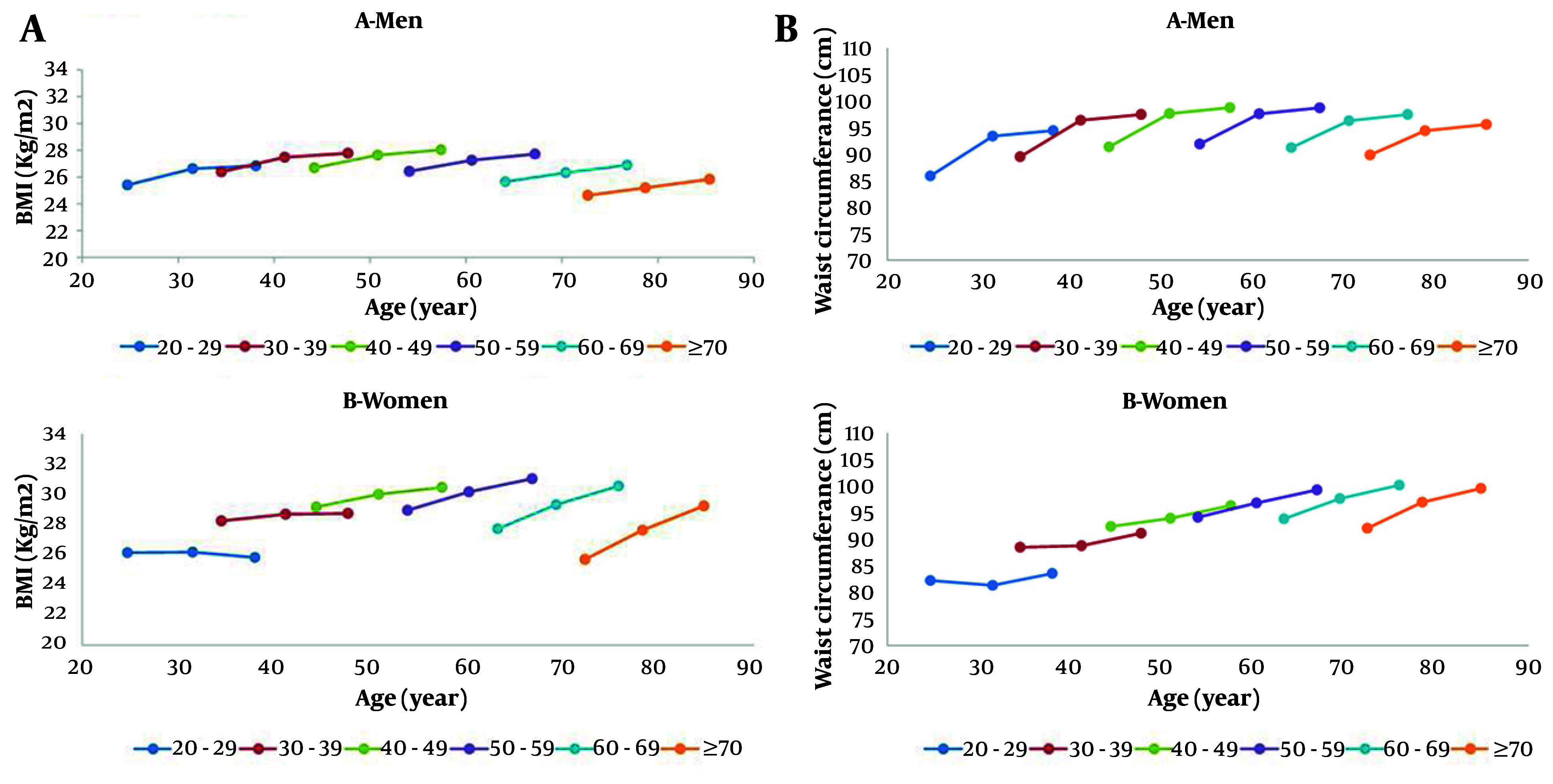
A, Trends of body mass index (BMI); and B, waist circumference in different age cohorts of study population across phases in I, III, and V in men and women.

Finally, in a prospective investigation of 4,895 adults aged ≥ 20 years (2,024 men and 2,871 women), participants were categorized into twelve gender-specific age groups (six per gender), each separated by ten-year intervals, to examine the distinct effects of age and period on longitudinal changes in BMI and WC. Over 15 years of follow-up, both BMI and WC increased significantly in men and women across all age cohorts. Overall, participants exhibited age-related increases in BMI and WC throughout the study period. In men, the rise in BMI and WC was more accelerated in younger birth cohorts. This cohort effect was statistically significant for BMI at Phase III and for WC at Phases III and V, demonstrating a significant inverse relationship where later year of birth was associated with higher adiposity. Conversely, among women, the trends were different: Older generations showed sharper increases in BMI and WC at both Phases III and V. This indicates a significant positive link, meaning that women born earlier experienced a greater rise in these measures (Figure 1) (9).

The following longitudinal studies within the TLGS framework shift the focus to the pediatric population, elucidating the trends, incidence, and key predictors of obesity among children and adolescents. A longitudinal study, part of the TLGS, examined the trends in childhood obesity over a decade (1999 - 2011) among 1,406 Tehranian children aged 3 - 11 years at baseline. The overall prevalence of obesity increased from 5.5% in Phase I to 9.4% in Phase IV, peaking at 13.0% in Phase III. A similar increasing pattern was observed in both genders, with higher rates in boys than girls throughout the study. Generalized estimating equation (GEE) analysis revealed a significant increase in the relative risk (RR) of obesity from phase I to phase II (RR = 1.06), stabilization from phase II to phase III (RR = 1.01), and a slight decline from Phase III to Phase IV (RR = 0.96). Subgroup analyses indicated that younger children (aged < 7 years at baseline) experienced a more pronounced and sustained increase in obesity risk compared to older children (≥ 7 years). Additionally, children with obese parents consistently showed higher obesity rates across all phases. Parental education and smoking status did not significantly influence the obesity trend (10).

Another longitudinal cohort study, conducted within the TLGS framework, investigated the incidence and predictors of obesity in a Tehranian urban population of children and adolescents over a 10-year follow-up. Among 1,033 non-obese participants aged 7 - 11 years at baseline, the cumulative incidence of obesity was 17%, with a higher rate in boys (19.6%) than girls (14.5%). Children aged 7 - 9 years had a significantly greater obesity incidence (22%) compared to those aged 10 - 11 years (10.8%). Cox proportional hazard models identified several strong predictors for developing obesity: Being overweight at baseline (HR = 14.93), having a waist circumference at or above the 90th percentile (HR = 5.05), the presence of childhood metabolic syndrome (HR = 2.77), and having an obese father (HR = 2.69) or mother (HR = 3.04). The study identifies early childhood as a critical period for anti-obesity interventions, particularly targeting children under 7 who are overweight, have a high WC, metabolic syndrome (MetS), or obese parents (11).

### 3.2. Body Composition and Its Assessment

Complementary evidence from a series of cross-sectional analyses within the TLGS cohort details body composition parameters in adults, exploring their association with age, gender, and cardiometabolic risk. In a cross-sectional study of 2,970 adults (1,511 men, mean age: 42.2 ± 13.2 years; and 1,459 women, mean age: 41.9 ± 11.7 years), participants were categorized into five age groups to evaluate body composition parameters by gender and age using bioelectrical impedance analysis (BIA). Obesity indices were significantly higher in women, whereas skeletal muscle mass (SMM) and fat-free mass (FFM) were significantly greater in men. Both SMM and FFM declined markedly after the age of 50 years in both genders. In men, obesity indices increased significantly from the 20 - 29 to 30 - 39-year age group, while in women, significant increases were observed between the 30 - 39 and 40 - 49-year, as well as between the 40 - 49 and 50 - 59-year age groups. The fat mass ratio (fat mass/SMM) exhibited two peaks in both genders: after ages 30 and 50 in men, and after ages 40 and 50 in women. A strong positive correlation was observed between BMI and percentage body fat, with correlation coefficients of r = 0.823 in women and r = 0.768 in men (12).

In further cross-sectional analysis of adults aged ≥ 20 years from the TLGS, Heidari Almasi et al. evaluated the comparative utility of BMI and percent body fat (PBF) measured by BIA for predicting cardiometabolic risk. The study determined population-specific cutoffs for PBF and BMI to identify metabolic syndrome but found no significant superiority of PBF over BMI in predictive accuracy, as assessed by area under the receiver operating characteristic curve, for most risk factors. Indeed, logistic regression analyses indicated that BMI was an equal or better predictor than PBF for the majority of cardiometabolic abnormalities in both sexes. The authors concluded that despite its limitations, BMI remains a simple and effective tool for assessing cardiometabolic risk in clinical and public health practice, with PBF offering little additional advantage for this purpose in the general adult population (13).

In another cross-sectional study including 860 participants (420 men, mean age: 69.0 ± 7.4 years; and 440 women, mean age: 67.0 ± 6.3 years), the overall prevalence of low skeletal muscle mass was 16.4%, with a higher prevalence observed in men than in women (20.24% vs. 12.73%, respectively). Low skeletal muscle mass was positively associated with older age, being a man, smoking, low levels of physical activity, and elevated HDL concentrations. Conversely, higher values of BMI, body weight, height, waist and hip circumference, and serum triglyceride levels appeared to protect against low muscle mass (14).

### 3.3. Obesity, Body Composition, and Cardiometabolic Disease Associations

Subsequent prospective analyses from the TLGS cohort shift the focus to the long-term cardiometabolic outcomes of early-life adiposity, specifically examining the link between growth trajectories, childhood obesity phenotypes, and adult subclinical atherosclerosis, assessed by carotid intima-media thickness (cIMT). Over an 18-year prospective study of 1,265 individuals aged 3 - 18, participants with a high-increasing BMI trajectory had significantly greater cIMT in adulthood than those with a low-stable trajectory. No significant association was found for the moderate-increasing BMI trajectory. In contrast, both moderate- and high-increasing WC trajectories, relative to the low-stable group, were significantly associated with elevated cIMT (> 90th percentile for gender and age groups). Even after controlling for initial BMI, these relationships retained their significance. The odds ratios for high cIMT were 3.24, 1.92, and 3.29 for the high-increasing BMI, moderate-increasing WC, and high-increasing WC trajectories, respectively (15).

In another prospective cohort study involving 875 adolescents (454 men, mean age: 13.3 ± 2.1 years; and 421 women, mean age: 13.5 ± 2.2 years) followed for a median of 18.2 years, significant gender-specific associations were observed between early-life anthropometric indices and cIMT in adulthood. Among men, all anthropometric indicators — including BMI, WC, WHR, and waist-to-height ratio (WHtR) — were associated with high cIMT (> 90th percentile for age- and gender-specific groups). However, after adjustment for corresponding adulthood anthropometric measures, only WC and WHR maintained significant associations. Notably, in early adolescent boys (aged 10 - 14 years), all anthropometric indices remained significantly associated with elevated cIMT even after multivariate adjustment. Among male participants, the discriminatory accuracy of anthropometric measures, quantified by the AUC, varied from 0.576 for WHtR to 0.632 for WC, with no statistically significant differences among them. In women, linear regression analysis revealed that higher WC and WHtR values were significantly associated with adult cIMT; however, these associations were attenuated to null after accounting for adult anthropometric measures (16).

Additionally, in a longitudinal analysis of the TLGS that tracked participants from childhood to young adulthood (ages 3 - 18 to 20 - 40 years), the cumulative burden and developmental trajectories of BMI and blood pressure were investigated for their association with subclinical atherosclerosis, measured by cIMT. The cumulative burden of BMI and diastolic blood pressure (DBP) was significantly linked to early signs of atherosclerosis (high cIMT) in young adulthood. For every standard deviation increase in BMI burden, the odds of high cIMT increased by 35%, and for DBP burden, by 27%. Furthermore, the rate of increase in BMI (linear slope) throughout childhood and elevated levels of BMI and DBP specifically during adolescence (ages 13 - 18 and 11 - 17, respectively) were identified as significant risk periods for the development of increased cIMT (17).

Furthermore, in a prospective study from the TLGS, Tasdighi et al. (2022) (18) assessed the relation between childhood obesity phenotypes and early adulthood subclinical atherosclerosis, measured by cIMT. The study included 1,220 Iranian children and adolescents (51.7% boys) with a mean age of 10.9 years at baseline, who were followed for a median of 18 years. Based on their BMI and metabolic health status, participants were classified into one of four groups: Metabolically healthy normal weight (MHNW), metabolically unhealthy normal weight (MUNW), metabolically healthy obese (MHO), and metabolically unhealthy obese (MUO). A significant ascending trend in cIMT values was observed across the phenotype spectrum, from MHNW to MUO (P for trend < 0.001). After accounting for gender, age, family history of CVD, and smoking, only the MUO phenotype was linked to a significantly higher risk of high cIMT, more than doubling the risk (Relative Risk [RR] = 2.13). However, this link was no longer significant after accounting for the participants' BMI in adulthood. This suggests that the increased risk associated with the MUO phenotype is largely explained by higher body weight in later life. The study underscores that the combination of obesity and metabolic unhealthiness in childhood poses the highest risk for subclinical atherosclerosis in early adulthood, but this risk is largely contingent upon the persistence of obesity into adult life (18).

Further longitudinal investigations from the TLGS cohort assess the role of childhood and adolescent adiposity as a predictor for the development of major metabolic disorders in later life, including MetS and type 2 diabetes. In a prospective cohort study of 888 children aged 6–12 years (390 boys and 498 girls) with a mean follow-up duration of 6.6 years, the cumulative incidence of MetS was reported to be 10.7%. After controlling for gender, age, and family history, the odds ratios (ORs) of MetS for BMI and WC z-scores were both 2.6. The areas under the receiver operating characteristic (ROC) curves for BMI and WC z-scores were 0.73 (95% CI: 0.68 – 0.79) and 0.72 (95% CI: 0.67 – 0.78), respectively, with no statistically significant difference between the two indicators. The analysis identified optimal predictive thresholds for MetS as a BMI of 16.5 kg/m² in boys and 16.3 kg/m² in girls, and a WC of 57.5 cm for boys and 56.5 cm for girls (19).

In another longitudinal cohort investigation, followed for a median of 14.9 years, Piri et al. investigated the association between childhood BMI and the development of dysglycemia (prediabetes or type 2 diabetes) in early adulthood. The study followed 1,290 normoglycemic Iranian children from the TLGS, with a mean baseline age of 7.7 years, into young adulthood (mean age 22.6 years). While unadjusted analyses showed that children with overweight/obesity had a higher incidence of dysglycemia, following adjustment for age, sex, parental history of diabetes, and the children's baseline cardiometabolic profile (including obesity, hypertension, waist circumference, blood pressure, and lipids), this association was attenuated and lost statistical significance. Accordingly, elevated BMI in childhood was not an independent risk factor for dysglycemia in early adulthood, suggesting that the apparent risk is mediated by other familial and concurrent metabolic factors (20).

Finally, in an analysis from the TLGS, Yaghoubpour et al. (2021) evaluated the longitudinal relation between adolescent obesity phenotypes and the incidence of T2DM in early adulthood. The analysis followed 2,306 Iranian adolescents (mean age 15.1 years) for a median of 15.5 years. Subjects were classified into metabolically healthy/unhealthy and normal weight/obese phenotypes. The study revealed that while MHO was not associated with an enhanced T2DM risk, MUO adolescents of both sexes and MUNW boys exhibited a significantly higher hazard. Notably, after adjustment for adulthood BMI, the risk associated with the MUNW phenotype persisted only in boys, suggesting that underlying metabolic disturbances in normal-weight male adolescents may predispose them to future T2DM independent of adult weight status (21).

Complementary prospective studies within the TLGS cohort quantify the impact of adult adiposity on the incidence of major non-communicable diseases, specifically examining its potential effect in the development of type 2 diabetes and chronic kidney disease. In a prospective cohort study with a mean follow-up duration of 3.6 years, the influence of overweight/obesity on the type 2 diabetes incidence was quantified. Among 4,728 participants (1,922 men and 2,716 women), 182 individuals (3.8%) developed T2DM during follow-up. The incidence rates were 1.4%, 3.6%, and 7.8% among participants with normal weight, overweight, and obesity, respectively. Compared with individuals with normal BMI, the adjusted odds ratios (ORs) for incident diabetes were 1.76 for overweight and 3.54 for obesity. After adjusting for age, triglycerides, and systolic blood pressure, and family history of T2DM, the population-attributable risk (PAR) was 23.3% for overweight and 37.1% for obesity. When stratified by gender, the PARs were 7.8% for overweight and 26.6% for obesity in men, compared to 35.3% and 48.3% in women, respectively (22).

Using data from the TLGS, Ghazy et al. (2024) (23) tracked 8,697 Iranian adults over 15 years to investigate the link between chronic kidney disease (CKD) incidence and different levels of obesity severity and duration. The study innovatively used cumulative metrics — Cumulative Excess Weight (CEW) and Cumulative Excess Waist Circumference (CEWC) — to capture the combined effect of the degree and duration of general and central adiposity. The findings revealed that the accumulation of general obesity (CEW) was significantly associated with an increased risk of incident CKD in both men (HR = 1.155) and women (HR = 1.105). However, the association for cumulative central adiposity (CEWC) was significant only in men (HR = 1.074), with no significant association observed in women overall. A sensitivity analysis indicated that the risk associated with both CEW and CEWC became more pronounced in individuals aged 50 years and older. This study underscores that the long-term burden of obesity is a critical risk factor for CKD, with notable sex-specific differences, particularly concerning fat distribution (23).

### 3.4. Obesity Phenotypes

Building upon its pioneering foundation, the TLGS cohort now leverages its long-term follow-up data to specifically investigate the CVD risk of different obesity phenotypes. This unique investigation aims to resolve the central debate in the field: Whether metabolic health status or adiposity itself is the primary driver of cardiovascular risk. The findings from this endeavor provide critical empirical data that resonate with the recent paradigm shift in defining obesity, moving beyond BMI to a clinical framework based on adiposity-related organ dysfunction, as proposed by the global Commission on Clinical Obesity (2).

**Figure 2.**
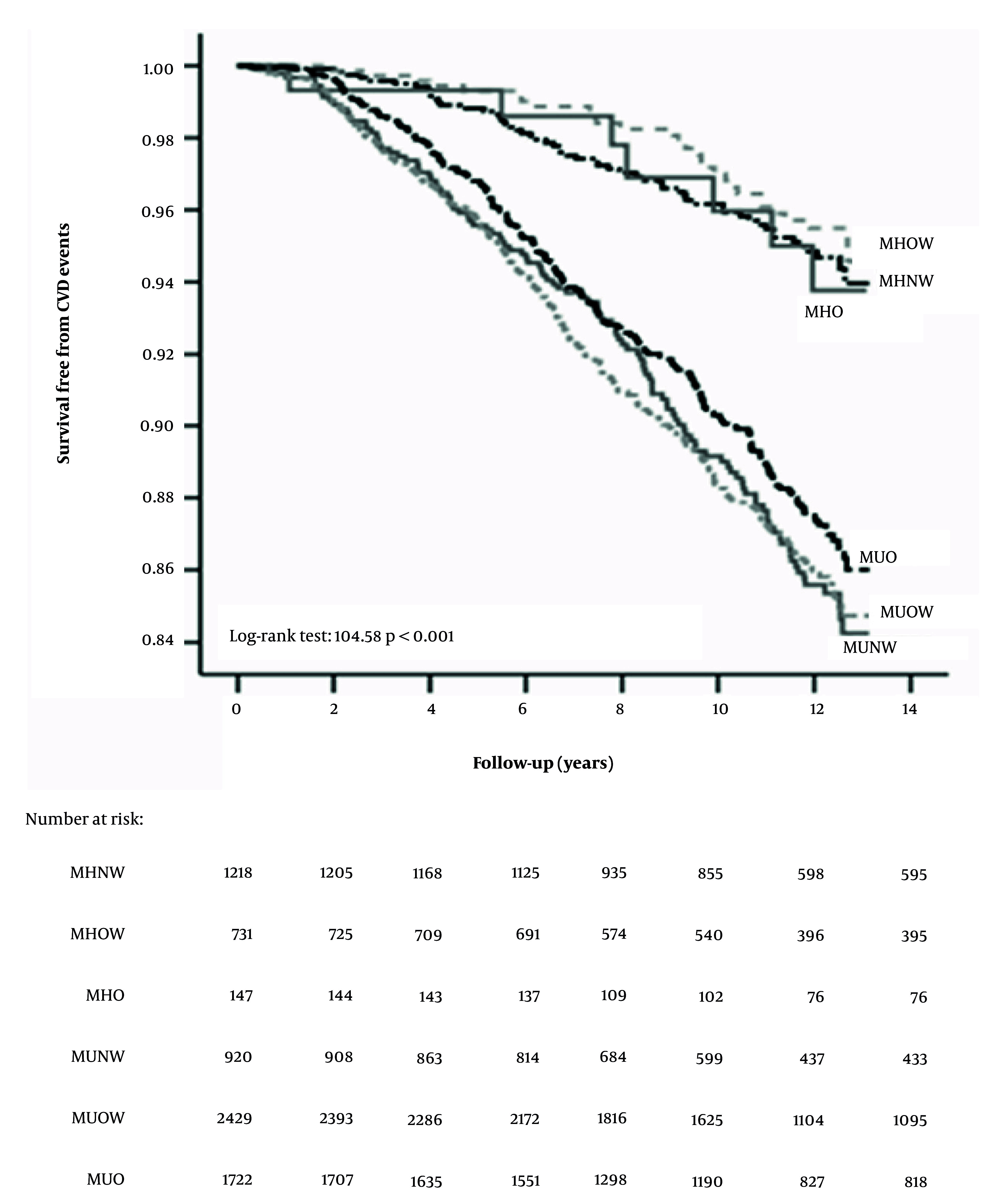
Kaplan-Meier curves for cumulative survival free from cardiovascular events as a function of obesity phenotypes according to body mass index and metabolic health.

In a 12-year follow-up study of 7,167 adults from the TLGS, Mirzaei et al. investigated the CVD risk associated with different obesity phenotypes, defined by BMI and metabolic health. The results demonstrated that metabolic health status was the primary determinant of CVD risk (24). Compared to the MHNW reference group, neither metabolically healthy overweight (MHOW) (adjusted HR 1.22, 95% CI 0.73 – 2.04) nor MHO (adjusted HR 1.74, 95% CI 0.68 – 4.44) individuals had a significantly increased risk of CVD events. In stark contrast, all metabolically unhealthy phenotypes — regardless of being normal weight, overweight, or obese — showed a significantly elevated CVD risk. When using insulin resistance to define metabolic health, an increased risk was observed in most groups, but this association was attenuated after adjusting for other metabolic risk factors. The authors concluded that over a 12-year period, the MHO and MHOW phenotypes were not associated with increased CVD incidence, underscoring that metabolic health, rather than obesity per se, is the critical factor for cardiovascular risk stratification (Figure 2) (24). These results provide a population-level evidence base for the newly proposed category of "Pre-clinical Obesity." Individuals with MHO/MHOW phenotypes, despite excess adiposity, lack the clinical signs of metabolic dysfunction that would define "Clinical Obesity," which aligns with their lower short-to-mid-term CVD risk in our findings.

Kokabeh et al. (2025) (25), in another prospective TLGS cohort study, analyzed the relationship of obesity phenotypes with incident CVD over an 18-year follow-up. Their methodology incorporated both conventional time-invariant and more nuanced time-varying Cox proportional hazards models. This study involved 7,118 Iranian adults (average age 46.6 years; 53.9% female). Participants were classified into six distinct phenotypes based on their BMI and metabolic health status: MHNW, MHOW, MHO, metabolically unhealthy normal weight (MUNW), metabolically unhealthy overweight (MUOW), and MUO. A total of 1,083 incident CVD events were recorded. Results from both analytical approaches consistently showed that metabolically healthy individuals, regardless of their BMI category, were not at an increased risk of CVD. In contrast, all metabolically unhealthy phenotypes exhibited a significantly elevated risk, with hazard ratios increasing across BMI categories in the time-invariant model (MUNW: HR = 1.85; MUOW: HR = 2.75; MUO: HR = 3.26) and similarly in the time-varying model. The study underscores that metabolic health status, rather than BMI alone, is a critical determinant of CVD risk, and underscores the significance of regular metabolic screening for individuals with overweight/obesity to prevent CVD (26).

To delineate the specific risks of abdominal obesity phenotypes, TLGS researchers have further stratified the population by abdominal obesity phenotypes, systematically evaluating their distinct associations with incident CVD, type 2 diabetes, kidney function decline, and all-cause mortality. In a large cohort of Tehranian adults aged 30 and over, our researchers prospectively assessed how different types of abdominal obesity phenotypes affect CVD risk over a median of 10 years. The study, which defined abdominal obesity using national WC cut-offs and metabolic health using Joint Interim Statement criteria, found that 23.5% of the abdominally obese population was categorized as "metabolically healthy abdominal obese" (MHAO). Crucially, after multivariable adjustment, individuals with the MHAO phenotype exhibited a significantly enhanced risk of CVD events (HR: 1.64; 95% CI: 1.09 – 2.47) compared to the metabolically healthy non-abdominal obese reference group. This finding provides a critical nuance to the earlier discussions on obesity phenotypes. It demonstrates that while general obesity without metabolic dysfunction (the MHO phenotype) may not confer significant short-to-medium-term CVD risk, the specific presence of abdominal adiposity — even in the absence of other metabolic abnormalities (the MHAO phenotype) — is a potent and independent risk factor for cardiovascular disease. This highlights that the location of fat storage is a critical determinant of risk, with abdominal fat being particularly pathogenic. This pattern of elevated risk was consistent across all unhealthy phenotypes and was also confirmed when insulin resistance was used to define metabolic status. The authors concluded that abdominal obesity is not harmless, even without other metabolic issues, and its prevention and management are crucial for reducing CVD risk (26). This finding is particularly significant in the context of the new clinical obesity framework. It suggests that abdominal obesity itself, captured by WC, may be an early marker of adiposity-related organ dysfunction (in this case, cardiovascular system), potentially placing many individuals with the MHAO phenotype on the spectrum towards "Clinical Obesity," even in the absence of full metabolic syndrome.

In another population-based cohort study of the TLGS with a median follow-up of 12 years, the risk of all-cause mortality was evaluated across different abdominal obesity phenotypes among 8,804 adults aged ≥ 30 years. Participants were categorized based on WC (using national cut-offs: ≥ 89 cm for men, ≥ 91 cm for women) and metabolic health (defined as ≤ 1 metabolic syndrome component excluding WC). The MHAO phenotype, which comprised 12.8% of the total population and 23.4% of those with abdominal obesity, did not show a significantly increased risk of all-cause mortality compared to the metabolically healthy non-abdominal obese (MHNAO) reference group after multivariate adjustment (HR: 1.35, 95% CI: 0.89 – 2.03). In contrast, both metabolically unhealthy phenotypes — whether abdominally obese or not — exhibited significantly elevated mortality risks. These findings suggest that, over a 12-year period, MHAO may not independently increase all-cause mortality risk. However, the authors caution that the analysis may have been underpowered and note that longer follow-up or assessment of other outcomes (e.g., cardiovascular events) may be necessary to fully understand the long-term risks associated with this phenotype (27).

Furthermore, in a 12-year follow-up study of 7,982 non-diabetic adults from the TLGS, Salehinia et al. investigated the risk of T2DM incidence across different abdominal obesity phenotypes, defined by WC and metabolic health. The results demonstrated that both metabolic unhealth and abdominal obesity independently contributed to diabetes risk (28). Compared to the MHNAO reference group, the metabolically healthy abdominal obese (MHAO) phenotype was associated with a significantly enhanced risk of diabetes in women (adjusted HR 1.68, 95% CI 1.08 – 2.6) and a borderline significant increase in men (adjusted HR 1.5, 95% CI 1.0 – 2.36). As expected, the metabolically unhealthy abdominal obese (MUAO) group had the highest risk (adjusted HR 2.3 in women and 1.59 in men). Notably, even metabolically unhealthy individuals without abdominal obesity (MUNAO) were at higher risk in women. Hence, abdominal obesity, even in the presence of metabolic health, is a significant risk factor for developing diabetes, highlighting the need for preventive strategies targeting WC regardless of an individual's current metabolic profile (28).

Furthermore, a 20-year prospective cohort study from the TLGS, involving 8,731 participants, revealed a clear hierarchy in CKD risk across obesity-metabolic phenotypes. After full adjustment for confounders, the MUO phenotype carried the highest risk (adjusted HR 1.63, 95% CI 1.42 – 1.87), followed by the metabolically unhealthy non-obese (MUNO) phenotype (adj. HR 1.49, 95% CI 1.33 – 1.67) and the MHO phenotype (adj. HR 1.35, 95% CI 1.18 – 1.54), using the metabolically healthy non-obese (MHNO) group as the reference. The findings underscore that metabolic health state is a more potent driver of CKD risk than obesity alone, as evidenced by the significant hazard in the MUNO group (29).

A key line of investigation within the TLGS has focused on the dynamic nature and long-term stability of MHO phenotypes, with a series of longitudinal studies examining the predictors of its incidence, the factors driving the transition to metabolically unhealthy states, and the associated cardiovascular risks over time. In a 10-year longitudinal analysis of the TLGS that tracked 916 metabolically healthy abdominally obese (MHAO) adults, the natural course and stability of this phenotype were investigated. The study found that a significant proportion of MHAO individuals transitioned to an unhealthy metabolic state over the decade, with 43.3% losing their metabolic health and 42.1% developing full MetS. Moreover, the study found that certain metabolic markers measured at the start of the study could significantly predict which individuals would undergo this transition. After adjustment for confounders, hypertriglyceridemia, low high-density lipoprotein cholesterol (HDL-C), and a higher homeostasis model assessment of insulin resistance (HOMA-IR) were independent risk factors for losing metabolic health, highlighting that underlying dyslipidemia and insulin resistance drive the deterioration in this putatively "healthy" population (30).

**Figure 3.**
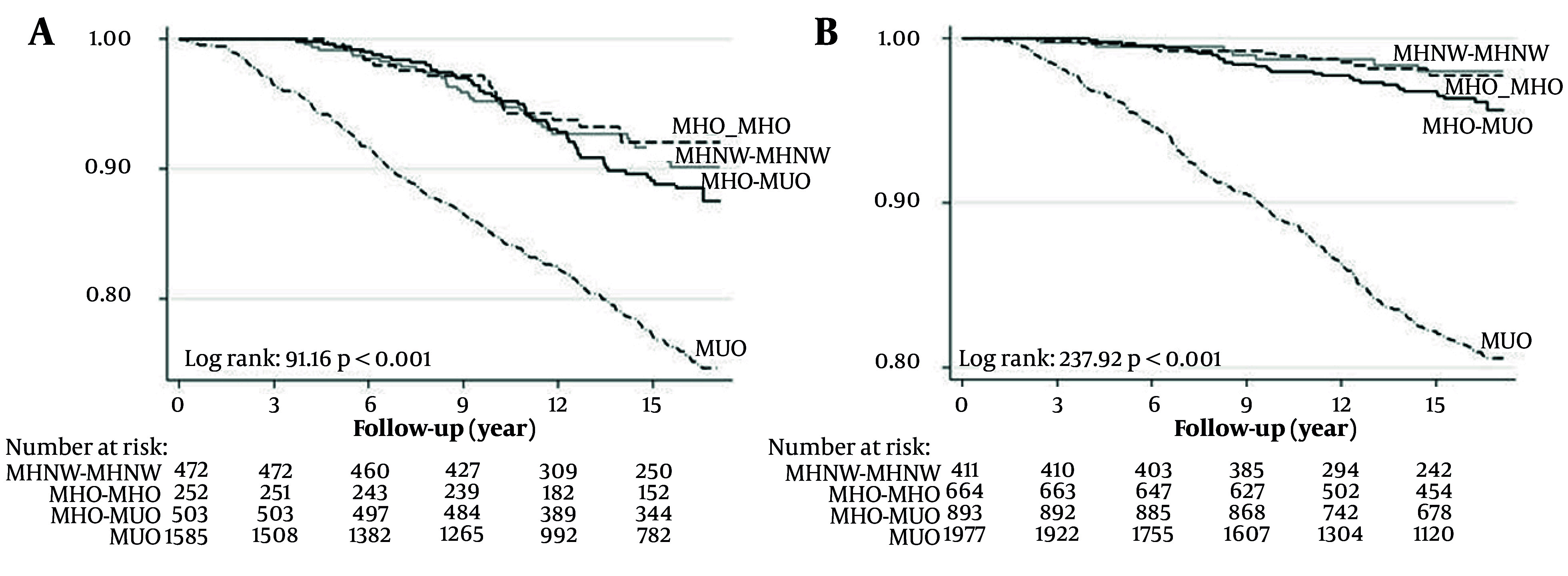
Kaplan-Meier curves for cumulative survival free from cardiovascular events as a function of obesity phenotypes according to body mass index and metabolic health in each obesity phenotype. A, male; B, female.

In another long-term prospective study within the TLGS, Hosseinpanah et al. (2020) investigated the risk of CVD associated with the transition from metabolically healthy overweight/obesity in a large population of Iranian adults with 20 years of age or older, with a median follow-up of 15.9 years (31). Their findings revealed that the MHO phenotype is dynamic, with a significant proportion transitioning to metabolically unhealthy status. Critically, while the persistent MHO phenotype was not associated with enhanced risk of CVD in either sex, the transition from MHO to metabolically unhealthy obesity significantly increased CVD risk in men (HR = 1.55), but not in women. This underscores that the primary CVD risk in MHO individuals may be driven by the progression to MetS, and it highlights a significant sexual dimorphism in this risk trajectory, indicating that obesity itself is not a risk factor if metabolic health is maintained (Figure 3) (31). In addition, briefly, Khalili and co-workers (29) claimed that the persistence of metabolically unhealthy states conferred the greatest risk, while transitions from a healthy to an obese-but-metabolically-healthy phenotype were associated with a lower risk, highlighting the dynamic nature of these phenotypes and the potential influence of exposure duration.

Hosseinpanah et al. (2014) conducted a decade-long prospective cohort study as part of the TLGS to examine the natural history of Metabolic Syndrome in a cohort of 438 adults who were obese but metabolically healthy at baseline (31). The study found a high cumulative incidence of MetS of 44.0% over the 10-year follow-up. After adjusting for other factors, the study found that high blood pressure, high triglycerides, low HDL cholesterol, and insulin resistance were all significant, independent predictors of MetS in this group. A critical finding was that WC was not an independent predictor. This suggests that the factors leading to MetS in MHO individuals are different from those in the general population. The authors concluded that a combination of HDL-C, triglycerides, and fasting blood sugar had a predictive power for MetS similar to all five standard MetS components (32).

In a longitudinal study investigating the stability of the MHO phenotype, Jahromi et al. followed 2,525 initially MHO adults from the TLGS over 15 years to assess the impact of long-term adiposity exposure on the transition to a MUO state. The study introduced the novel concepts of CEW and cumulative excess waist circumference (CEWC), which quantify the aggregate severity and length of general and abdominal adiposity over time. The results revealed a significant gender-specific association: A higher burden of cumulative general and central adiposity was strongly linked to an increased risk of MHO to MUO transition among women (e.g., fully adjusted HR for the highest CEWC quartile: 1.83, 95% CI 1.53 – 2.19). In men, however, neither CEW nor CEWC showed a significant link to metabolic deterioration. Therefore, the MHO phenotype is often transient, particularly in women, and highlights that the long-term accumulation of excess weight, especially around the waist, is a critical driver of metabolic decline, with important implications for sex-specific prevention strategies (33).

In a long-term TLGS analysis stratified by gender, Mousapour et al. (2021) evaluated whether wrist circumference could predict the MHO to MUO transition. The study followed 1,130 Iranian adults (309 men, 821 women) with a baseline MHO status for a median of 15.5 years. The results demonstrated a high rate of transition to MUO, affecting 87.1% of men and 77.5% of women. In initial, unadjusted analyses, a larger waist circumference was linked to a significantly higher risk of metabolic deterioration in both genders. However, following multivariable adjustment for confounders including age, BMI, and WC, wrist circumference remained an independent predictor of the transition only in women, with those in the highest tertile having a 34% higher risk (HR = 1.34) compared to the lowest tertile. The findings establish wrist circumference as a straightforward, inexpensive, and sex-specific anthropometric measure that can aid in stratifying risk among MHO individuals, with particular clinical utility for identifying high-risk women (34).

In alignment with our analysis of risk factors for predictors of transition of obesity phenotypes, we similarly explored the predictors for the dynamics in a longitudinal analysis of the TLGS. Accordingly, in a longitudinal analysis of the TLGS, Eftekharzadeh et al. investigated predictors of incident obesity phenotypes by following a cohort of 3,489 initially non-obese (BMI < 30 kg/m²), metabolically healthy Iranian adults (mean baseline age ~35 years) for a median of 13.4 years. Among the individuals who developed obesity, 36.6% were classified as MHO. The study identified distinct predictors for the MHO phenotype compared to MUO. Specifically, female gender, a higher baseline BMI, and elevated HDL-C levels were independent correlates of progressing to MHO. Conversely, older age, larger baseline WC, a greater increase in WC over time, and higher DBP were significant predictors for developing the MUO phenotype. The authors concluded that while central adiposity was a critical determinant, female gender was the strongest predictor, underscoring the importance of gender and fat distribution in determining the metabolic trajectory of individuals who become obese (35).

### 3.5. Defining Anthropometric Cut-offs in Tehranian Population

Further TLGS investigations have aimed to refine anthropometric risk assessment by establishing and validating population-specific cut points for BMI, WC, and body fat percentage to optimize the prediction of long-term cardiovascular risk and mortality.

In a large prospective cohort study comprising 7,412 participants (3,344 men, mean age: 47.4 ± 13.7 years; and 4,068 women, mean age: 43.2 ± 12.3 years) followed over 18 years, sex-specific WC thresholds were identified across different BMI categories (< 25, 25 – 30, and > 30 kg/m²) in relation to CVD events and all-cause mortality. For CVD outcomes, the WC cutoffs were 82, 95, and 103 cm in men and 82, 89, and 100 cm in women. Corresponding thresholds for all-cause mortality were 88, 95, and 103 cm in men and 83, 90, and 99 cm in women (36).

In a cross-sectional analysis of 212 healthy adults aged 20 - 30 years (89 men and 123 women), reference values for body composition were established based on the 95th percentile of PBF assessed by BIA in young reference groups. The mean body fat mass was 16.6 ± 4.9 kg in men and 31.1 ± 5.4 kg in women, with the lowest PBF observed between the ages of 20 and 30 years. The PBF cutoff points were determined to be 22.5% for men and 38.7% for women (37).

The predictive utility of different childhood BMI criteria for adult cIMT was evaluated in a prospective cohort of 1,295 individuals followed from childhood (age 3) to adulthood over 18 years. Participants were classified as having normal weight, overweight, or obesity using the definitions from the World Health Organization (WHO), the Centers for Disease Control and Prevention (CDC), the International Obesity Task Force (IOTF), and a locally adapted IOTF reference. The prevalence of overweight and obesity varied by classification system. Considering the WHO, CDC, local IOTF, and international IOTF criteria, overweight rates were 15.4%, 11.5%, 16.3%, and 14.1%, while obesity rates were 6.6%, 8.5%, 7.7%, and 5.0%, respectively. Across all classification systems, individuals in the obese group had significantly greater cIMT than those in the normal-weight group. Regression analyses demonstrated that the international IOTF criteria exhibited the strongest predictive ability for adulthood cIMT, followed by the local IOTF and WHO references, whereas the CDC criteria showed the lowest discriminatory capacity (38).

This work on refining anthropometric cut-offs directly supports the new clinical obesity paradigm's emphasis on using improved adiposity metrics for risk stratification. Establishing which measures and thresholds best predict future subclinical organ damage (such as increased cIMT) is a crucial step in identifying individuals with "Pre-clinical Obesity" who are at high risk of progressing to "Clinical Obesity" and its complications.

## 4. Conclusions

This review synthesizes a quarter-century of evidence from the TLGS on the obesity epidemic in Iran. The findings reveal alarming and sustained increases in both general and abdominal obesity prevalence among Tehranian adults and children, establishing this trend as a pervasive public health challenge that cuts across all sociodemographic strata. The TLGS has been instrumental in defining population-specific anthropometric thresholds and providing critical insights into the complex relationships between obesity phenotypes and a spectrum of cardiometabolic diseases. Key evidence demonstrates that abdominal obesity, even in the absence of concurrent metabolic abnormalities (the MHAO phenotype), is not a benign condition and carries a significantly elevated risk for cardiovascular disease and type 2 diabetes. The studies consistently highlight the particular vulnerability of the MUNW phenotype and underscore that metabolic health status often proves to be a more critical determinant of long-term cardiovascular risk than body mass index alone. Furthermore, research on childhood and adolescent obesity reveals that early-life adiposity, especially when it persists into adulthood, is a key driver of subclinical atherosclerosis in later life. A pivotal contribution of the TLGS has been its longitudinal perspective, revealing the dynamic nature of obesity phenotypes. The finding that a significant proportion of individuals with MHO transition to an unhealthy state over time underscores the transient nature of this condition and the importance of proactive monitoring. These findings strongly align with the new clinical obesity framework proposed by the 2025 Lancet Commission. The progression from "Pre-clinical Obesity" (akin to the MHO or MHAO phenotypes) to "Clinical Obesity" (manifested as MUO or the development of cardiometabolic diseases) is precisely the trajectory observed in our cohort. The longitudinal design has also enabled the examination of cumulative obesity exposure, showing that the aggregate burden of excess weight, particularly central adiposity, is a strong predictor of incident chronic kidney disease and metabolic deterioration, with notable sex-specific patterns observed. For instance, the burden of cumulative adiposity was a more potent predictor of metabolic deterioration in women, while wrist circumference emerged as a novel, gender-specific predictor for this transition.

From a public health and clinical perspective, these findings carry profound implications. They provide robust, population-level validation for the ongoing paradigm shift beyond simple weight classification towards a more integrated assessment that includes metabolic health, fat distribution, and the historical trajectory of an individual's adiposity, as championed by the new clinical obesity definition. Public health strategies must be dual-pronged: First, prioritizing population-wide primary prevention to curb the rising tide of obesity, particularly among the youth; and second, implementing targeted secondary prevention for high-risk phenotypes, such as individuals identified with "Pre-clinical Obesity" (e.g., MHAO) to prevent progression to "Clinical Obesity," and those who are metabolically unhealthy at any BMI. Despite its significant contributions, this review is subject to limitations, primarily the focus on an urban Iranian population, which may limit the generalizability of findings to rural settings or other ethnicities. Nevertheless, the lessons learned from this transitioning population are highly relevant to many low- and middle-income countries undergoing similar epidemiological shifts. These findings collectively emphasize that effective strategies must address both the prevention of weight gain across the lifespan and the early identification and management of adverse metabolic profiles, regardless of an individual's current weight status.

Future research stemming from the TLGS and similar cohorts should focus on elucidating the underlying mechanisms behind the observed sex differences, exploring the role of novel biomarkers and body composition metrics in risk stratification, and most importantly, designing and evaluating the effectiveness of tailored interventions for the high-risk phenotypes identified through this long-term cohort study. The TLGS was established with the ultimate goal of developing cost-effective prevention and control strategies for NCDs. While the primary focus of this review has been on the epidemiological and etiological insights generated over a quarter-century, the study's findings have directly informed public health action in several ways. The population-specific anthropometric cut-offs and the identification of high-risk phenotypes (such as MHAO and MUNW) provide a scientific basis for targeted screening and resource allocation, which is a fundamental principle of cost-effectiveness. Furthermore, the TLGS itself incorporated a community-oriented lifestyle intervention program, demonstrating the feasibility of such approaches in this population. Future translational research, including formal cost-effectiveness analyses of interventions tailored to the high-risk groups identified in this cohort, is a critical next step to fully realize the study's founding mission.

The quarter-century of data from the TLGS provides several unique strengths and novel contributions to the field of obesity research. As one of the longest-running prospective cohorts in the Middle East, its key strengths include its large, population-based sample, long-term follow-up with high retention rates, serial detailed phenotyping, and the unique context of a population undergoing rapid nutritional and epidemiological transition. These features have enabled several distinctive insights that are less evident in cohorts from Western or high-income countries. These include: (1) documenting the alarming speed and trajectory of the obesity epidemic in a transitioning population; (2) establishing the dynamic nature and sex-specific predictors of metabolically healthy obesity phenotypes over the very long term; (3) quantifying the risk of abdominal obesity independent of metabolic health, a finding that strongly aligns with emerging clinical frameworks; and (4) pioneering the concept of cumulative adiposity burden (CEW, CEWC) for predicting long-term metabolic and renal outcomes. Despite these strengths, our findings should be interpreted considering certain limitations. The study population is urban Iranian, which may affect the generalizability of the specific prevalence estimates and cut-off points to rural populations or other ethnicities. Furthermore, while extensive, the data are observational, and residual confounding cannot be entirely ruled out.
